# An Investigation of ZZ/ZW and XX/XY Sex Determination Systems in North African Catfish (*Clarias gariepinus*, Burchell, 1822)

**DOI:** 10.3389/fgene.2020.562856

**Published:** 2021-01-05

**Authors:** Dung Ho My Nguyen, Thitipong Panthum, Jatupong Ponjarat, Nararat Laopichienpong, Ekaphan Kraichak, Worapong Singchat, Syed Farhan Ahmad, Narongrit Muangmai, Surin Peyachoknagul, Uthairat Na-Nakorn, Kornsorn Srikulnath

**Affiliations:** ^1^Laboratory of Animal Cytogenetics and Comparative Genomics (ACCG), Department of Genetics, Faculty of Science, Kasetsart University, Bangkok, Thailand; ^2^Special Research Unit for Wildlife Genomics (SRUWG), Department of Forest Biology, Faculty of Forestry, Kasetsart University, Bangkok, Thailand; ^3^Department of Botany, Kasetsart University, Bangkok, Thailand; ^4^Department of Fishery Biology, Faculty of Fisheries, Kasetsart University, Bangkok, Thailand; ^5^Department of Aquaculture, Faculty of Fisheries, Kasetsart University, Bangkok, Thailand; ^6^Center for Advanced Studies in Tropical Natural Resources, National Research University-Kasetsart University, Kasetsart University, Bangkok, Thailand; ^7^Center of Excellence on Agricultural Biotechnology (AG-BIO/PERDO-CHE), Bangkok, Thailand; ^8^Omics Center for Agriculture, Bioresources, Food and Health, Kasetsart University (OmiKU), Bangkok, Thailand; ^9^Amphibian Research Center, Hiroshima University, Higashihiroshima, Japan

**Keywords:** fish, SNP, recombination, aquaculture, transposable element

## Abstract

An investigation of sex-specific loci may provide important insights into fish sex determination strategies. This may be useful for biotechnological purposes, for example, to produce all-male or all-female fish for commercial breeding. The North African catfish species, *Clarias gariepinus*, has been widely adopted for aquaculture because its superior growth and disease resistance render the species suitable for hybridization with other catfish to improve the productivity and quality of fish meat. This species has either a ZZ/ZW or XX/XY sex determination system. Here, we investigate and characterize these systems using high-throughput genome complexity reduction sequencing as Diversity Arrays Technology. This approach was effective in identifying moderately sex-linked loci with both single-nucleotide polymorphisms (SNPs) and restriction fragment presence/absence (PA) markers in 30 perfectly sexed individuals of *C. gariepinus*. However, SNPs based markers were not found in this study. In total, 41 loci met the criteria for being moderately male-linked (with male vs. female ratios 80:20 and 70:30), while 25 loci were found to be moderately linked to female sex. No strictly male- or female-linked loci were detected. Seven moderately male-linked loci were partially homologous to some classes of transposable elements and three moderately male-linked loci were partially homologous to functional genes. Our data showed that the male heterogametic XX/XY sex determination system should co-exist with the ZZ/ZW system in *C. gariepinus*. Our finding of the co-existence of XX/XY and ZZ/ZW systems can be applied to benefit commercial breeding of this species in Thailand. This approach using moderately sex-linked loci provides a solid baseline for revealing sex determination mechanisms and identify potential sex determination regions in catfish, allowing further investigation of genetic improvements in breeding programs.

## Introduction

Aquaculture plays an important role in global food production (Belton and Thilsted, 2014; Loring et al., 2019). However, the availability of land and water resources for aquaculture is currently limited, while susceptibility to new diseases is increasing (Ayyam et al., 2019; Lebel et al., 2019). This has facilitated continuous improvements in breeding programs by cross-breeding between different species to generate new hybrid varieties that can tolerate high stocking densities (Dunham and Masser, 2012; Zhou et al., 2018). Clariid catfish (*Clarias* spp.) are commonly distributed in freshwater from the River Orange in South Africa to the Nile in North Africa, as well as in parts of Asia (Israel, Syria, Southern Turkey, and Southeast Asia) (Skelton and Teugels, 1992; Teugels and Adriaens, 2003; Na-Nakorn et al., 2004a). With good flesh quality (taste and firmness), the bighead catfish (*Clarias macrocephalus*, Günther, 1864) is one of the most economically important freshwater fish in Southeast Asia (Günther, 1864; Teugels et al., 1999). However, it is difficult to develop sustainable breeding management programs because of the low growth rate and high susceptibility to disease (Jarimopas et al., 1988; Na-Nakorn et al., 1993; Senanan et al., 2004). The North African catfish species (*C. gariepinus*, Burchell, 1822) was introduced for hybridization with bighead catfish to improve the productivity and quality of the fish meat (Burchell, 1822; Nukwan et al., 1990). A hybrid was developed by crossing artificially male North African catfish and female bighead catfish (Senanan et al., 2004). This F_1_ hybrid catfish exhibits a rapid growth rate and has a high disease resistance, which has led to the propagation of these hybrids in the aquaculture market. They now represent more than 90% of catfish production in Thailand (Na-Nakorn et al., 2004a). The F_1_ hybrids are physically more vigorous than either parental species, but their mass production has been limited by reproductive failure (Koolboon et al., 2014; Ponjarat et al., 2019). Ponjarat et al. (2019) asserted that hybrid dysgenesis between the bighead catfish and North African catfish is caused by karyotypic and genomic differences, resulting in meiotic arrest and subsequent apoptosis of gametocytes. However, the F_1_ hybrid can occasionally cross-breed in captivity, whereby the female hybrids are fertile, with the potential to produce large numbers of backcross progeny that shows fertility and low embryo mortality, whereas the male hybrids are sterile (Na-Nakorn et al., 2004b; Abol-Munafi et al., 2006). Sex hormones have been applied in an attempt to promote spermatogenesis, but the F_1_ male hybrids have always remained sterile (Abol-Munafi et al., 2006). The complexity of sex determination interaction between the two species might influence differences in fertility between F_1_ male and female hybrids (Ponjarat et al., 2019).

The North African catfish species, *C. gariepinus* has been widely adopted for aquaculture within and outside its native ranges (Van der Bank, 1998; Dai et al., 2011; Wachirachaikarn and Na-Nakorn, 2019), and is considered the tropical catfish species best suited to aquaculture (Clay, 1979; Okonkwo and Obiakor, 2010), leading to the application of *C. gariepinus* for cross-breeding with other clariid catfish (Rahman et al., 2013). They have been extensively distributed around the world (Vitule et al., 2006; Na-Nakorn and Brummentt, 2009). The farming of *C. gariepinus* is rapidly expanding due to its superior growth and disease resistance against fungi, viruses and bacteria (Akoll and Mwanja, 2012; Taukhid et al., 2015). *C. gariepinus* that carried the MHC-II marker showed better growth and were significantly different from other catfish (Suprapto et al., 2017). Moreover, they are also important for biological research such as genome manipulation (Bongers et al., 1995; Galbusera et al., 2000; Erondu et al., 2011; Nwachi and Dasuki, 2017). However, the problematics of taxonomy and systematics are complex and need thorough revision with inclusion of populations from different geographic origins (Teugels, 1982, 1984, 1986). Historically, several stocks of *C. gariepinus* have been introduced to Thailand (Wachirachaikarn and Na-Nakorn, 2019), and genetic diversity of a few has been identified significantly different between Thailand and Nigeria (Falaye et al., 2011; Wachirachaikarn and Na-Nakorn, 2019). Therefore, a proper understanding of the sex determination system of *C. gariepinus* in Thailand is necessary to assist in breeding programs of this species and its hybrid.

**FIGURE 1:**
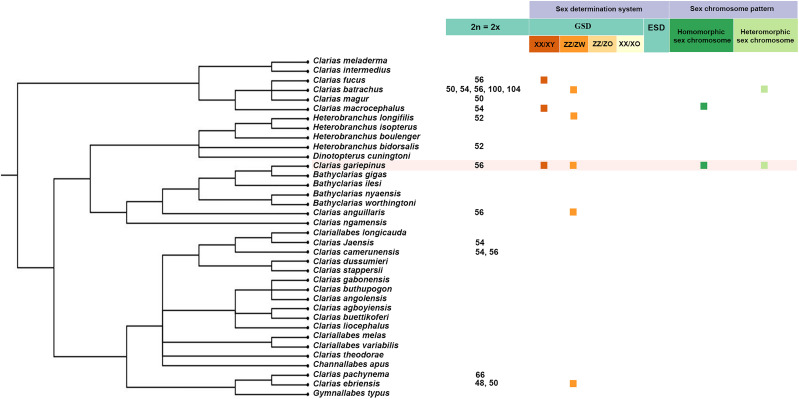
Phylogenetic tree showing number of chromosomes and sex determination system of the Clariidae family (Ozouf-Costaz et al., 1990; Teugels et al., 1992; Na-Nakorn, 1995; Bakhoum, 1996; Liu et al., 1996; Eding et al., 1997; Pandey and Lakra, 1997; Kovács et al., 2001; Eyo and Effiong, 2005; Malla and Ganesh, 2009; Okonkwo and Obiakor, 2010; Awodiran et al., 2015; Maneechot et al., 2016).

The family of air-breathing or labyrinth catfish (Clariidae) is one of the largest among the Siluriformes (Burgess, 1989). Clariid fish comprise 14 genera with 115 species found in India, Syria, Southern Turkey, Southeast Asia, and Africa. Most have the highly conserved karyotype with diploid chromosome number (2n) ranging from 48 to 56, with the exception of *C. pachynema* (2n = 66), and one population of *C. batrachus* (2n = 104) (Jianxun et al., 1991; Eyo and Effiong, 2005; Malla and Ganesh, 2009; Maneechot et al., 2016). These findings show that *C. gariepinus* phylogenetically exhibits both the XX/XY and ZZ/ZW system (Figure 1). However, significant variation in fish sex chromosome systems has been recorded, not only among closely related species (such as in tilapias, ricefishes, or sticklebacks; Takehana et al., 2008; Ross et al., 2009; Ser et al., 2010; Cnaani, 2013) but also between different populations of the same species (e.g., *Eigenmannia virescens* and *Ancistrus* cf. *dubius*; Almeida-Toledo et al., 2001; Mariotto et al., 2004;, Mariotto and Miyazawa 2006; Fernandes et al., 2019). Recent genotyping using next-generation sequencing, such as Diversity Arrays Technology (DArTseq^TM^) developed by Diversity Arrays Technology Pty Ltd., (Canberra, ACT, Australia), is an effective method for the identification of sex-linked loci in non-model species. The variability in SNP loci generates presence/absence polymorphism in restriction sites (so-called PA markers) and these may facilitate the identification of divergent genomic regions present in one sex only, thus pointing to a putative region of suppressed recombination on a presumed sex chromosome. To identify the sex determination system in the *C. gariepinus*, we applied DArTseq^TM^ in captive-bred individuals scored with phenotypic sex. The mapped DArTseq^TM^ sequences were then used to search for homologies with other ‘model’ teleosts (Japanese rice fish: *Oryzias latipes*, zebrafish: *Danio rerio*, and Japanese pufferfish: *Takifugu rubripes*), and amniotes (chicken: *Gallus gallus*). Our findings also provide novel insights into the evolutionary history of sex determination in catfish.

## Materials and Methods

### Specimens and DNA Extraction

Fifteen male and fifteen female individuals of North African catfish (*C. gariepinus*) were collected with weight of 1.7–2.0 kg and length of 35–40 cm. The sampled individuals were randomly picked from a large breeding stock to avoid the probability of high incidence of siblings in our pool. The sex of each individual was identified by external morphology and internal examination of gonadal morphology (Kitano et al., 2007; Esmaeili et al., 2017; Ponjarat et al., 2019). Hence, the sex of all specimens obtained from the Department of Aquaculture, Faculty of Fisheries, Kasetsart University (Bangkok, Thailand) was determined to avoid potentially wrong sexing due to sex reversal. The fins were removed for DNA extraction. Animal care and all experimental procedures were approved by the Animal Experiment Committee, Kasetsart University (Approval No. ACKU61-SCI-026), and conducted in accordance with the Regulations on Animal Experiments at Kasetsart University. Whole genomic DNA was extracted following the standard salting-out protocol as previously described (Supikamolseni et al., 2015). The quality of extracted DNA was evaluated by gel electrophoresis, and samples with high-molecular weight DNA were stored at −20°C until required for DArTseq^TM^ library construction.

### DArT Sequencing and Genotyping

A detailed description of the DArTseq^TM^ methodology can be found in Jaccoud et al. (2001). The method often produces 69 base pairs (bp) long sequences. Genotyping of multiple loci was performed using DArTseq^TM^ (Diversity Arrays Technology Pty Ltd., Canberra, Australian Capital Territory, Australia) for SNP loci and *in silico* DArT (PA of restriction fragments in the representation; PA loci) to determine the candidate sex-specific loci between male and female individuals. Approximately 100 ng of DNA from each sample was used for the development of DArTseq^TM^ arrays. DNA samples were subjected to digestion/ligation reactions as described by Kilian et al. (2012) and digested with *Pst*I and a second restriction endonuclease (*Sph*I). Ligation reactions were performed using two adaptors: a *Pst*I compatible adaptor consisting of an Illumina flow-cell attachment sequence, primer sequence, and a unique barcode sequence; and a *Sph*I compatible adaptor consisting of an Illumina flow-cell attachment region. Ligated fragments were then amplified by PCR using the following parameters: initial denaturation at 94°C for 1 min, followed by 30 cycles of 94°C for 20 s, 58°C for 30 s, and 72°C for 45 s with a final extension step at 72°C for 7 min. Equimolar amounts of amplification products from each individual were pooled and subjected to Illumina’s proprietary cBot^1^ bridge PCR followed by sequencing on the Illumina HiSeq 2000 platform. Single read sequencing was run for 77 cycles.

Sequences were processed using proprietary DArTseq^TM^ analytical pipelines (Ren et al., 2015). Initially, the HiSeq 2000 output (FASTQ file) was processed to filter poor-quality sequences. Two different thresholds of quality were applied. For the barcode region (allowing parsing of sequences into specific sample libraries), we applied stringent selection (minimum Phred pass score of 30, minimum pass length percentage 75). For the remainder of the sequence, relaxed thresholds were applied (minimum Phred pass score 10, minimum pass length percentage 50). Approximately 2,000,000 sequences per individual were identified and used in marker calling. Finally, identical sequences were combined into “fastqcoll” files that were used in the secondary proprietary pipeline (DArTsoft14) for SNP and PA loci calling. To this end, we used the “reference-free” algorithm implemented in DArTsoft14. The sequence clusters were then parsed into SNP and *in silico* DArTseq^TM^ markers utilizing a range of metadata parameters derived from the quantity and distribution of each sequence across all samples in the analysis. Multiple libraries of the same individual were included in the DArTseq^TM^ genotyping process, enabling reproducibility scores to be calculated for each candidate marker. Outputs by DArTsoft14 were then filtered on the basis of reproducibility values, average count for each sequence (sequencing depth), balance of average counts for each SNP allele, and call-rate (proportion of samples for which the marker was scored).

### Marker Selection and DArT Sequencing Analysis

Sex-specific loci were derived from the analysis of SNP co-dominant markers and PA as dominant markers. The SNP data were coded as “0” for the reference allele homozygote (the most common allele), “1” for the SNP allele homozygote, “2” for the heterozygote, or “–” as the double null/null allele homozygote (absence of a SNP fragment in the genomic representation). The PA data were coded as “1” for presence, “0” for absence, or “–” for putative heterozygosity. For sex-linked markers in an XX/XY sex-determination system, reference alleles are expected to be located on the X-chromosome. Here, “SNP alleles” were those that showed polymorphism relative to the reference allele. In an XX/XY system, SNP alleles should be associated with the Y sex chromosome, and located in or near to the male-determination region if the allele is tightly Y-specific. If the two sex chromosomes recombine, SNP alleles should occasionally appear on the X chromosome. Some males might then be homozygous for SNP alleles at particular loci, with females being heterozygous, exhibiting a copy of the SNP allele but the probability of a female being homozygous for a SNP allele should be low. For evaluation of loci associated with an XX/XY system, loci with female homozygosity frequencies for the reference allele of at least 70% were retained, whereas those with homozygosity at the SNP allele at no more than 30% and heterozygosity at no more than 30% were discarded. For males, loci with homozygosity frequencies for the reference allele of at most 30% and heterozygosity of at least 70% were retained. However, this allowed sex-linked loci to show a higher degree of SNP allele homozygosity for males if recombination occurred. For PA markers, loci that had restriction fragments sequenced in at least 70% of males and not sequenced in at least 70% of females were selected. The SNP and PA loci sequenced for 80%, 90%, and 100% of males were also included in a separate dataset. Loci passing the 100% filtering criterion were designated as perfectly sex-linked, whereas those passing at 70–90% were called moderately sex-linked loci. An opposite similar approach was carried out for targeting loci with a ZZ/ZW system.

Calculation of the Hamming distance was performed to determine the number of combined loci between male and female individuals for pairwise differences in SNP and PA loci using the “rdist” function of R version 3.5.1 statistical software (R Core Team, 2019). Heatmaps were plotted using the ggplot2 R package (R Core Team, 2019). The Hamming distance represents the number of pairwise differences between all individuals across all loci. To examine the genetic association between each locus and phenotypic sex assignment from the SNP and PA loci, the Cochran-Armitage trend test (CATT) was performed using the “catt” function of R version 3.5.1 with the HapEstXXR package (R Core Team, 2019; Sven and Klaus, 2019). The CATT results were similar to those of a chi-square test to assess whether the proportion of different genotypes followed the null expectation. Polymorphism information content (PIC), as an index for evaluation of the informativeness of SNP and PA loci, was calculated for each locus and ranged from 0 (fixation of one allele) to 0.5 (frequencies of the two alleles are equal).

### Estimation of Expected Sex-Linked Markers

The probability of candidate loci showing random associations with sex under a small sample size was estimated (Lambert et al., 2016). The formula *P*_*i*_ = 0.5*^*n*^* describes the probability of a locus being perfectly sex-linked by chance, where *P* is the probability for a given locus, *i* is sex-linked, 0.5 is the probability that either a female is homozygous or a male is heterozygous at a given locus, and *n* is the number of individuals sequenced at the locus. After sequencing, we multiplied *P* by the number of high-quality SNPs produced to estimate the number of SNPs expected to exhibit a perfectly sex-linked pattern by chance.

### Comparison of Potential Sex-Linked Loci

We scored all candidate loci, the frequency of analyzed loci was not strictly (100%) sex-linked. A three-sample chi-square for PA loci and heterozygosity for SNP loci assuming unequal variances (based on results from the descriptive statistics) was performed to determine whether these three groups were significantly different from each other using the chi-square test with the R package “stats” for PA loci, and the Kruskal-Wallis test using the R package “stats” and the Nemenyi test using the R package “PMCMR” for SNP loci, to determine the mean heterozygosity and standard deviation for each (Gruber and Georges, 2019). All candidate loci were plotted against each individual using the ‘glPlot’ function in the R package ‘dartR’ (Gruber and Georges, 2019; R Core Team, 2019).

### Homology Searching

For all sex-linked loci that reached our criteria and had statistically significant associations with phenotypic sex, a Basic Local Alignment Search Tool (BLAST) search was performed to investigate the homologies of the sex-linked SNP/PA loci against a selection of available teleost fish genomes (Japanese rice fish: *O. latipes*) (Accession No. GCF_002234675.1) (Kasahara et al., 2007), zebrafish: *D. rerio* (Accession No. GCA_000002035.4) (Broughton et al., 2001), Japanese pufferfish: *T. rubripes* (Accession No. GCA_901000725.2) (Elmerot et al., 2002), amniote reference genomes (chicken: *G. gallus*) (Accession No. AADN00000000.5) (International Chicken Genome Sequencing Consortium, 2004). We selected the aforementioned species as representative reference genomes in the homology search analysis due to the availability of their high-quality gene annotations and near complete up-to-date assemblies. The BLAST homology search was performed in two rounds. First we aligned sex-linked loci against the reference fish genome, and then we mapped the homologous genes to further clarify their location on sex chromosomes of high-quality annotated genomes representing vertebrate and invertebrate species. Using the BLASTn program, sex-linked loci were used to search the NCBI database^2^ and RepBase version 19.11 (Bao et al., 2015) (Genetic Information Research Institute, http://www.girinst.org/). This is a specialized nucleotide sequence collection for repeated or other significant sequences that only reports E-values lower than 0.05 and query coverage with similarity of more than 50%.

## Results

### Determination of Sex-Linked Loci in North African Catfish

**FIGURE 2:**
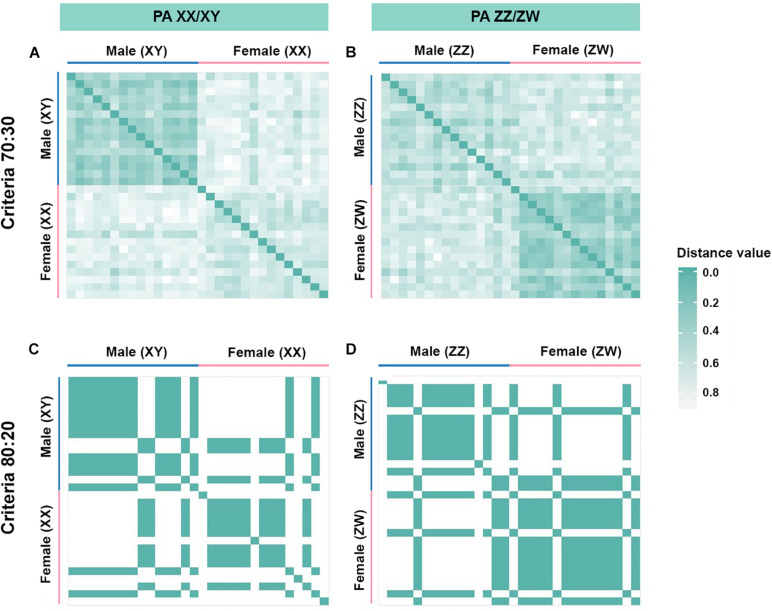
Hamming distance between male and female individuals of African catfish (*Clarias gariepinus*). **(A)** restriction fragment presence/absence (PA) loci with the criterion 70:30 (male:female), **(B)** PA loci with the criterion 30:70 (male:female), **(C)** PA loci with the criterion 80:20 (male:female), **(D)** PA loci with the criterion 20:80 (male:female).

**FIGURE 3:**
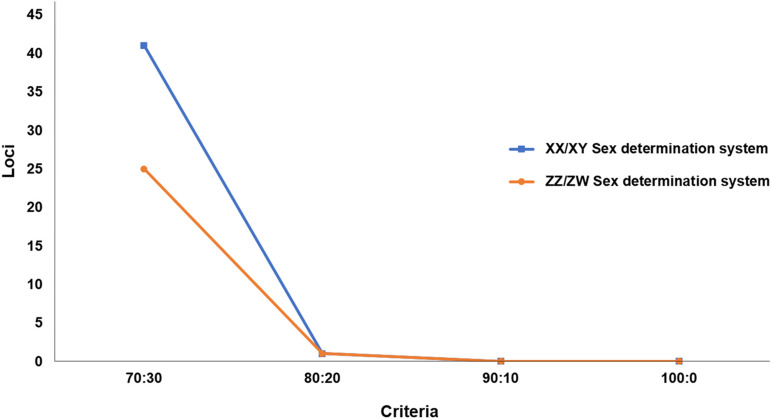
Graph showing number of loci for different hypotheses of sex determination systems after filtering with different criteria. X-axis indicates the number of loci and Y-axis shows the filter criteria.

We sequenced 42,752 SNP loci and 118,118 PA loci. PIC values ranged from 0.49 to 0.50 for all loci. To determine whether XX/XY or ZZ/ZW sex chromosomes drive sex determination in *C. gariepinus*, we compared a number of SNP and PA after filtering with a gradually varying set of criteria. For the ZZ/ZW type, filtering using the criterion of 30:70 male:female presented only 25 PA loci as moderately female-linked and no SNP loci. Proportional pairwise Hamming distance between males and females using moderately sex-linked PA loci showed within-sex distances of 0.563 ± 0.011 in males and 0.403 ± 0.014 in females, and showed between-sex distances of 0.675 ± 0.007 for PA loci. The CATT results verified a significant locus association with phenotypic sex for 25 PA (χ2 = 5.07–11.63; *p* < 0.001) loci. Moreover, 20:80 male:female yielded only one PA loci as moderately female-linked and no SNP loci. Hamming distance between males and females using moderately sex-linked PA loci showed within-sex distances of 0.543 ± 0.049 in males and 0.343 ± 0.047 in females. Between-sex distances showed 0.707 ± 0.03 PA loci. CATT verified a significant locus association with sex phenotype for one PA (χ2 = 9.07; *p* < 0.001) locus (Figure 2). No SNP or PA loci associated with females was found for the criteria of 10:90 and 0:100 male:female (Figure 3, Table 1). Chi-square tests showed that 30:70 and 20:80 filtering criteria indicated no significant differences in males (χ2 = 1.2226 × 10^–31^; *p* = 1) and females (χ2 = 1.0206 × 10^–31^; *p* = 1) for PA.

**TABLE 1:** DArT analysis of 15 males and 15 females of African catfish (Clarias gariepinus) (ZZ/ZW sex-determination type).

	30:70 male:female		20:80 male:female		10:90 male:female		0:100 male:female	
	PA^1^	SNP^2^	PA^1^	SNP^2^	PA^1^	SNP^2^	PA^1^	SNP^2^
Total number of DArT analyses	118,118	42,752	118,118	42,752	118,118	42,752	118,118	42,752
Moderately sex-linked loci	25	-	1	-	-	-	-	-
Overall mean distance between males and females	0.675 ± 0.007	-	0.707 ± 0.03	-	-	-	-	-
Overall mean distance within females	0.403 ± 0.014	-	0.343 ± 0.047	-	-	-	-	-
Overall mean distance within males	0.563 ± 0.011	-	0.543 ± 0.049	-	-	-	-	-

*^1^Presence/Absence (PA) loci. ^2^Single nucleotide polymorphism (SNP) loci.*

**FIGURE 4:**
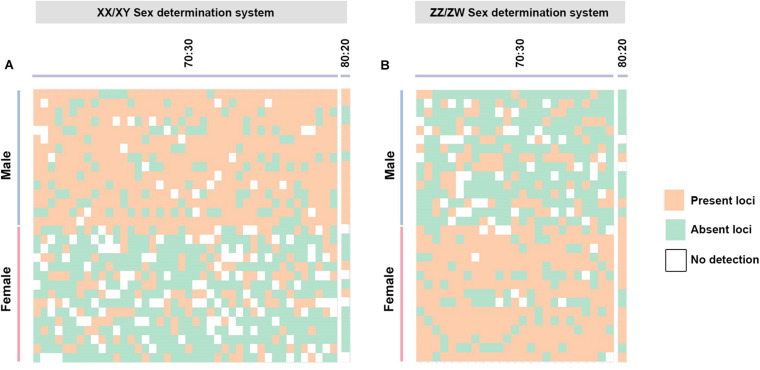
**(A)** Index of 41 moderately sex-linked loci with criterion of 70:30 (male:female) (XX/XY sex determination system) and **(B)** index of 25 moderately sex-linked loci with criterion of 30:70 (male:female) (ZZ/ZW sex determination system) created using the ‘glPolt’ function in the R package ‘dartR.’ Orange indicates loci presence, green is indicative of loci absence, and white indicates null loci.

By contrast, for the XX/XY type, filtering using the criterion of 70:30 male:female yielded 42 PA loci as moderately male-linked loci and no SNP loci. Proportional pairwise Hamming distance between male and female African catfish using moderately sex-linked PA loci (under the null exclusive model) showed lower within-sex distances of 0.373 ± 0.01 in males and 0.624 ± 0.009 in females for PA loci. Between-sex distances showed 0.699 ± 0.006 for PA loci. CATT verified significant loci association with phenotypic sex for 41 PA (χ2 = 3.55–13.06; *p* < 0.001) loci. The criterion of 80:20 male:female yielded one PA locus as moderately male-linked. Hamming distance between males and females using moderately sex-linked PA loci showed within-sex distances of 0.343 ± 0.047 in males and 0.648 ± 0.047 in females, and between-sex distances of 0.773 ± 0.0028 for PAloci. CATT verified significant loci association with phenotypic sex for one PA (χ2 = 9.75; *p* < 0.001) locus (Figure 2). The criteria of 90:10 and 100:0 male:female did not show any PA locus as male-specific (Figure 3, Table 2). Chi-square tests revealed that the 70:30 and 80:20 filtering criteria produced no significant differences in males (χ2 = 4.5003 × 10^–32^; *p* = 1) and females (χ2 = 2.4989 × 10^–32^; *p* = 1) for PA. A glPlot revealed that the sample group showed greater similarity between sexes when moderately sex-linked loci were considered in both XX/XY and ZZ/ZW sex determination systems (Figure 4).

**TABLE 2:** DArT analysis of 15 males and 15 females of African catfish (*Clarias gariepinus*) (XX/XY sex-determination type).

	70:30 male:female		80:20 male:female		90:10 male:female		100:0 male:female	
	PA^1^	SNP^2^	PA^1^	SNP^2^	PA^1^	SNP^2^	PA^1^	SNP^2^
Total number of DArT analyses	118,118	42,752	118,118	42,752	118,118	42,752	118,118	42,752
Moderately sex-linked loci	41	-	1	-	-	-	-	-
Overall mean distance between males and females	0.699 ± 0.006	-	0.773 ± 0.0028	-	-	-	-	-
Overall mean distance within females	0.624 ± 0.009	-	0.648 ± 0.047	-	-	-	-	-
Overall mean distance within males	0.373 ± 0.01	-	0.343 ± 0.047	-	-	-	-	-

*^1^Presence/Absence (PA) loci. ^2^Single nucleotide polymorphism (SNP) loci.*

### Estimation of Expected Sex-Linked Markers

For a range of sample sizes and loci, a sample of 30 phenotypically sexed individuals is essential to minimize the probability of selecting less than one spurious sex-linked marker. The *P*_*i*_ probability that a single locus exhibited a perfectly sex-linked pattern by chance is 9.31 × 10^–10^. Considering that we analyzed the full dataset (no filtering) of 160,870 (including SNP and PA loci), this translates to an expected number of perfect sex-linked loci due to chance alone of 1.5 × 10^–4^.

### Homology of Putative Sex-Linked Loci

In terms of a ZZ/ZW sex chromosome system, female moderately sex-linked loci of *C. gariepinus* had sequence homology with Japanese rice fish (*O. latipes*), Japanese pufferfish (*T. rubripes*), and zebrafish (*D*. *rerio*) genomes on the basis of global BLAST analyses of NCBI databases. No sequence homology was found with the chicken genome (Supplementary Table 1). We found that 2 of 25 PA loci were homologous with putative genes: *PCDH2AB3* (E-values 2 × 10^–11^, Query Cover 100% and similarity 82.61%) and *DCTN4* (E-values 4 × 10^–8^, Query Cover 68% and similarity 89.58%) from *D. rerio* (Wu, 2005; Bayés et al., 2017) (Supplementary Table 2). Moreover, 4 of 25 PA loci had partial homology with transposable elements, comprising 3 loci with similarity to *Gypsy* families and 1 locus of retroelement *Rex1* (Supplementary Table 3). By contrast, for the XX/XY sex chromosome system, 3 out of 41 moderately male sex-linked PA loci of *C. gariepinus* had sequence homology with putative genes: *ADD3* (E-values 2 × 10^–4^, Query Cover 71% and similarity 81.63%) from *D. rerio* (Pasquier et al., 2016), *GUCD1* (E-values 0.038, Query Cover 56% and similarity 82.05%) from *T. rubripes* (Elmerot et al., 2002), and *DNTA* (E-values 0.003, Query Cover 52% and similarity 86.11%) from *G. gallus* (Bellott et al., 2017) (Supplementary Table 4), whereas 7 of 41 PA loci had partial homology with transposable elements consisting of 3 loci of *Gypsy*, 3 loci of *Tc1/mariner* families, and 1 locus of short interspersed nuclear elements (*SINEs*) (Supplementary Table 5). Moreover, one PA locus was matched with a non-coding region homologous to the chicken Z chromosome (GGAZ) (Supplementary Table 6).

## Discussion

Fish sex chromosomes are often homomorphic (i.e., cytologically indistinguishable) which hampers their analysis (Otake et al., 2010; Gammerdinger and Kocher, 2018). Such a condition, however, seems to facilitate frequent sex chromosome turnovers (Gammerdinger and Kocher, 2018; Ieda et al., 2018). The variation in fish sex chromosome systems has been recorded among closely related species (such as in tilapias, ricefishes, or sticklebacks; Takehana et al., 2008; Ross et al., 2009; Ser et al., 2010; Cnaani, 2013) and also between different populations of the same species (e.g., *Eigenmannia virescens* and *Ancistrus* cf. *dubius*; Almeida-Toledo et al., 2001; Mariotto et al., 2004; Mariotto and Miyazawa, 2006; Fernandes et al., 2019). Given these facts, the identification of sex-linked loci is important for a thorough elucidation of the sex chromosome origin and evolution in teleosts, on both inter-specific and inter-population levels (Herpin and Schartl, 2015; Kottler and Schartl, 2018). An intriguing situation has been found in *C. gariepinus*, where several reports differed in the type of detected sex chromosome systems, suggesting either XX/XY or ZZ/ZW constitution (Ozouf-Costaz et al., 1990; Bakhoum, 1996; Liu et al., 1996; Eding et al., 1997; Váradi et al., 1999; Okonkwo and Obiakor, 2010). Here, DArTseq^TM^ technology was applied to a large number of SNP/PA loci, allowing the prediction of sex-linked loci for *C. gariepinus* using a sample size of 30 individuals (15 males and 15 females). Many false-positive signals might be expected from such specimens due to their more diverse genetic background (Gamble et al., 2015); however, our data show the feasibility of our approach. The probability of one locus showing sex linkage spuriously was 9.31 × 10^–10^ from the full dataset (no filtering) of 160,870 loci (including SNP and PA loci), whereas the expected amount of perfect sex linkage was estimated to be 1.5 × 10^–4^; thus, identification of any sex-linked loci by chance seems unlikely. Our analysis reports the co-occurrence of male and female linked loci with a different proportion (70:30) across all the sampled specimens, suggesting that the XY and ZW systems can coexist inside the same fish individual. Our results indicate that sex chromosome turnover might still be in action in this species.

### Both ZZ/ZW or XX/XY Sex Chromosome Systems Co-exist in North African Catfish

**FIGURE 5:**
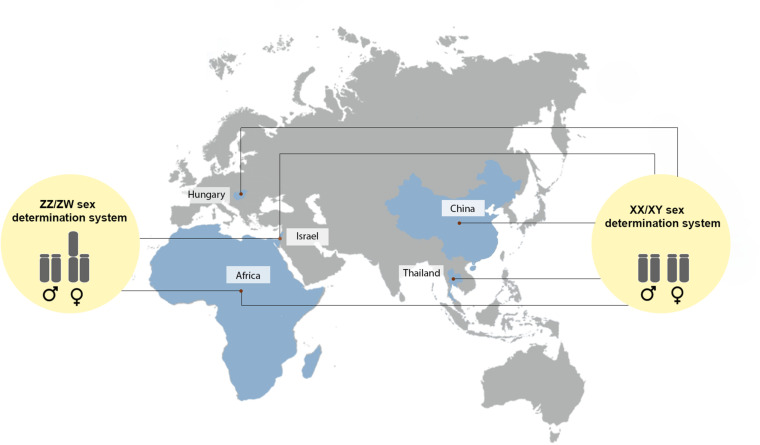
Population map indicating a male heterogamety (XX/XY) and female heterogamety (ZZ/ZW) belonging to different groups (Ozouf-Costaz et al., 1990; Teugels et al., 1992; Liu et al., 1996; Galbusera et al., 2000; Kovács et al., 2001; Maneechot et al., 2016; Ponjarat et al., 2019).

Most of the reported sex chromosome systems from Africa and Israel support the assumption that the ZZ/ZW system is the ancestral type for *C. gariepinus* (Ozouf-Costaz et al., 1990; Teugels et al., 1992; Eyo and Effiong, 2005). However, molecular marker, chromosome analysis, genome manipulation (both gynogenesis and androgenesis), and hormonal sex-reversal experiments with different sampled populations from Israel, Hungary and China have suggested a XX/XY system in *C. gariepinus* (Liu et al., 1996; Eding et al., 1997; Galbusera et al., 2000; Figure 5). Our study successfully identifies 41 SNP/PA loci as moderately male-linked loci, in contrast to 25 moderately female-linked loci. Based on the comparison between several teleost reference genomes, the loci were not all located in the same linkage group. One scenario might be that all loci are in the same linkage group for *C. gariepinus* but, just hypothetically, this chromosome pair was evolved by fusion. Comparison of moderately sex-linked loci between the two candidate systems showed that two female-linked loci had homology with putative protein-coding genes. One of these two loci was homologous to the *DCTN4* gene located on the sex chromosome in platypus (*Ornithorhynchus anatinus*) (Warren et al., 2008) (Supplementary Table 7). By contrast, three moderately male-linked loci were homologous to protein-coding genes, including *GUCD1*, which is located on the sex chromosome of the monarch butterfly (*Danaus plexippus plexippus*), tongue sole (*Cynoglossus semilaevis*), fruit fly (*Drosophila busckii*), and dog (*Canis lupus familiaris*) (Supplementary Table 8) (Lindblad-Toh et al., 2005; Chen et al., 2014; Gu et al., 2019; Renschler et al., 2019). However, in this study, moderately male-linked markers were found simultaneously with moderately female-linked markers in the same individuals, as found in cichlids (Roberts et al., 2016). This suggests that the XX/XY sex determination system might co-exist with ZZ/ZW system in the same individuals of *C. gariepinus*.

**FIGURE 6:**
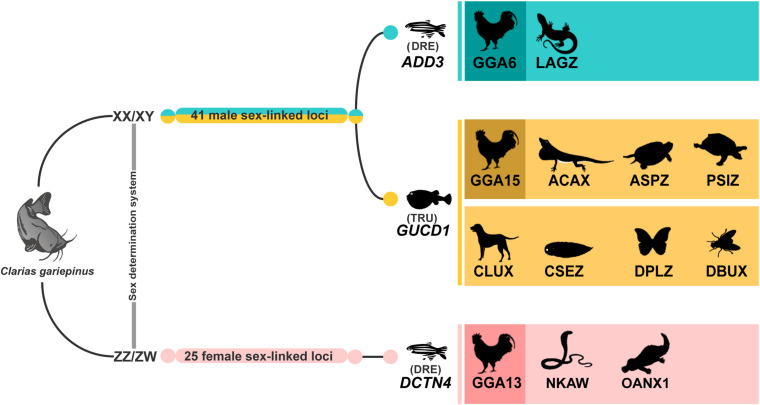
Moderately male-linked and female-linked loci of Africa catfish (*Clarias gariepinus*) showing partial homologies with putative genes: *ADD3* (*Danio rerio*) (Pasquier et al., 2016), *GUCD1* (*Takifugu rubripes*) (Elmerot et al., 2002) and *DCTN4* (*Danio rerio*) (Bayés et al., 2017). These genes were located on chromosomes of chicken (*Gallus gallus*, GGA) and other amniote sex chromosomes, such as sand lizard (*Lacerta agilis*, LAG), green anole (*Anolis carolinensis*, ACA), spiny softshell turtle (*Apalone spinifera*, ASP), Chinese softshell turtle (*Pelodiscus sinensis*, PSI), dog (*Canis lupus familiaris*, CLU), tongue sole (*Cynoglossus semilaevis*, CSE), and invertebrates, such as fruit fly (*Drosophila busckii*, DBU), monarch butterfly (*Danaus plexippus plexippus*, DPL), and female-specific loci of African catfish (*Clarias gariepinus*) showing homologies with chicken (*Gallus gallus*, GGA), platypus (*Ornithorhynchus anatinus*, OAN), Siamese cobra (*Naja Kaouthia*, NKA). Chromosomal locations of genes in the amniotes were obtained from comparative gene mapping (chromosome mapping via a cytogenetic technique) and whole genome sequencing as the following sources: GGA from Matsuda et al. (2005), ACA from Alföldi et al. (2011), LAG from Srikulnath et al. (2014), ASP from Badenhorst et al. (2013), PSI from Kawagoshi et al. (2009), CLU from Lindblad-Toh et al. (2005), DBU from Renschler et al. (2019), CSE from Chen et al. (2014), DPL from Gu et al. (2019), OAN from Veyrunes et al. (2008), and NKA from Singchat et al. (2018).

Genomic convergence has been detected by comparative cytogenetics studies in which unrelated sex chromosomes share sex chromosomal linkage homologies across distantly related species (Srikulnath et al., 2014, 2015; Ahmad et al., 2020; Koomgun et al., 2020; Laopichienpong et al., 2020; Singchat et al., 2020a,b). We found that a male-linked locus under the 70:30 criterion shared partial homology with *ADD3* which is localized on GGA6, which is further orthologous to sex chromosomes of sand lizard (*Lacerta agilis*). In addition, another male-linked 70:30 locus had a partial homology with *GUCD1* on GGA15 that is partially homologous to sex chromosomes of green anole (*Anolis carolinensis*), spiny softshell turtle (*Apalone spinifera*), and Chinese softshell turtle (*Pelodiscus sinensis*) (Figure 6; Kawagoshi et al., 2009; Alföldi et al., 2011; Badenhorst et al., 2013; Rovatsos et al., 2014; Srikulnath et al., 2014; Singchat et al., 2020a,b). Similar cases were also observed in which linkage homologies or small partial linkage homologies were conserved between teleost and amniote sex chromosomes (Kasahara et al., 2007; Cortez et al., 2014). Our results are only based on observations of certain PA loci showing homology to segments presented on some sex chromosomes of other amniotes or members of gene families, not exactly the identical gene. Moreover, lots of material between sex chromosomes and autosomes moves via transposition (in both directions); therefore, some genes can be independently co-opted to relocate to different linkage groups, with abundant evidence, typically in mammals (Mitchell et al., 1998; Uechi et al., 2002; Kuroiwa et al., 2010; Hughes et al., 2015). This could be further studied by other approaches such as whole genome sequencing and genome assembly. One moderately female-linked locus was observed in *C. gariepinus* corresponding to GGA13 and partially homologous to sex chromosomes of platypus (*Ornithorhynchus anatinus*) and W sex chromosome of Siamese cobra (*Naja kaouthia*) (Veyrunes et al., 2008; Singchat et al., 2018; Figure 6). Moderately sex-linked loci derived from 70:30 and 80:20 (male:female) filtering criteria indicate partial recombination between X and Y sex chromosomes. These loci are relatively close to potential male-determining regions but they are not intimately connected.

Although transposable elements can be dispersed on many chromosomes or even throughout the entire genome, large amplification of the elements are often found on sex chromosomes in many organisms (Ahmad et al., 2020; Koomgun et al., 2020; Laopichienpong et al., 2020; Yoshido et al., 2020). The presence of accumulated TEs leads to the establishment of heterochromatin, and possibly also to TE recombination between non-homologous copies. The activity of transposable elements could play an important role in sex chromosome differentiation through mechanisms of chromosomal breakage, deletion, and rearrangement (Chalopin et al., 2015; Dechaud et al., 2019). Seven out of 41 male-linked SNP/PA loci were identical to transposable elements such as *Gypsy*, *Mariner/Tc1*, and *SINE*, which are commonly distributed on sex chromosomes in Japanese rice fish (*O. latipes*) and half-smooth tongue sole (*C. semilaevis*) (Chen et al., 2014; Inoue et al., 2017).

### Same Species of North African Catfish at Different Localities Show Different Sex Determination Systems

Karyotypes of *C. gariepinus* from different localities of tropical Africa and Israel are very stable and exhibit the ZZ/ZW type (Ozouf-Costaz et al., 1990; Teugels et al., 1992; Bakhoum, 1996; Okonkwo and Obiakor, 2010). However, our analysis of *C. gariepinus* with sampled populations from Thailand indicates the presence of the XX/XY co-existing with ZZ/ZW type. Possibly, *C. gariepinus* has two different sex chromosome groups in Bangui, Central African Republic, West Africa, while other fish species such as the southern platyfish, *Xiphophorus maculatus*, exhibit a different mechanism of sex determination among diverse populations (Volff and Schartl, 2001). Similarly, both ZZ/ZW and XX/XY sex chromosome systems have been reported in the glass knifefish, *Eigenmannia virescens.* The ZW group is distributed in Mato Grosso do Sul State, Brazil, with the XY group in the Tietê River, Brazil (Almeida-Toledo et al., 2001; Fernandes et al., 2019), while *Ancistrus* cf. *dubius* revealed female heterogamety in the Paraguay River Basin, Brazil and male heterogamety in the Coxipó River, Brazil (Mariotto et al., 2004; Mariotto and Miyazawa, 2006). Another well-known example of ZW/XY co-occurrence has been reported in some fish species of cichlids such as *Oreochromis niloticus*, *Oreochromis aureus*, *Astatotilapia burtoni* and *Metriaclima pyrsonotus*. These species have a male heterogametic (XY) system and a female heterogametic (ZW) system on different linkage groups (Ser et al., 2010; Cnaani, 2013; Moore and Roberts, 2013; Conte et al., 2017; Gammerdinger and Kocher, 2018). The majority of the sex chromosome systems in clariid catfish show the ancestral karyotype with ZZ/ZW system (Agnèse et al., 1990; Volckaert and Agnèse, 1996). This suggests that the ZW group with differentiated sex chromosomes of *C. gariepinus* might be distributed in West Africa, whereas the turnover of sex chromosome systems might have occurred in ancestral homomorphic ZW sex chromosomes, resulting in the XY group. Historically, more than one stock of *C. gariepinus* has been introduced to Thailand but the origin of this population remains unknown (Wachirachaikarn and Na-Nakorn, 2019). Thus, it is necessary to identify potential geographic variation in sex linkage across these loci, and across boundary regions between the geographic groups, as well as examining the phylogenetic history for various populations of *C. gariepinus*. Moreover, it is very interesting to examine the mutual affinity of the two different systems of sex determination when they meet secondarily after divergence from each other. Specimen collection of *C. gariepinus* from Africa with the XY group is necessary to prove these hypotheses.

A distinct difference exists between growth rates of the two sexes in *C. gariepinus* (Henken et al., 1987; Falaye et al., 2011), and improvement of genome manipulation such as gynogenesis or androgenesis and hormone treatment procedures by early molecular sexing could be economically important for species aquaculture. Results of genome-wide SNP such as those from DArTseq^TM^ methodology provide sex-specific SNP/PA loci in moderately sex-linked loci for identifying sex determination and sex-linked markers, although this can be applied only narrowly across species (Lambert et al., 2016; Hill et al., 2018). The next phase of this study requires a PCR-based approach for sex identification to provide a genotypic tool for the practical sexing of individuals, allowing accurate detection of sex in populations through insight into the evolutionary history of the Y chromosome. However, failure of the PCR validation step has often been observed after DArTseq^TM^ or RADseq bioinformatics analysis (Gamble et al., 2015). This outcome might result from conserved regions in both sexes of sequences adjacent to sex-specific restriction sites (Gamble, 2016).

## Conclusion

We have presented an approach of genome-wide SNP used to identify a notable number of moderately sex-linked loci given the small portion of the genome involved. Results of moderately linked (and lacking sex-specific) markers in both male and female individuals show that the male heterogametic XX/XY sex determination system should co-exist with ZW system in the same individuals of *C. gariepinus*, indicating a probable ongoing transition between two sex chromosome systems. However, it remains unclear whether the location of large genomic regions between X-specific and Y-specific fragments is associated with differentiation of sex chromosomes and sex-determination regions. It might be possible that the two *C. gariepinus* populations exhibit different sex chromosome systems. Thus, the possibility exists of within-species sex chromosome turnover, which could be further tested by other methods such as genome assembly. Further analysis of unrelated individuals from the wild is also required to understand the dynamics of the sex determination system in this lineage. Chromosome mapping using fluorescence *in situ* hybridization (FISH) technique on sex-specific loci should be performed on both mitotic and meiotic chromosomes, while immunostaining of synaptonemal complexes may show regions of asynapsis on putative sex chromosomes. A high-quality whole genome assembly for North African catfish will further enhance our understanding of the sex-determination mechanism and lead to the development of more robust assays for genotypic sex, allowing a much greater degree of genetic improvement in the breeding management of North African catfish, bighead catfish, and their hybrids.

## Data Availability Statement

The full dataset and metadata from this publication are available from the Dryad Digital Repository. Dataset, https://doi.org/10.5061/dryad.prr4xgxj6.

## Ethics Statement

The animal study was reviewed and approved by Animal care and all experimental procedures were approved by the Animal Experiment Committee, Kasetsart University (Approval No. ACKU61-SCI-026).

## Author Contributions

DN, TP, and KS drafted the manuscript and conducted the experiments. DN, NL, EK, and KS conceived the ideas and designed the methodology. DN, EK, and KS participated in the data analysis. DN, TP, JP, NL, EK, WS, SA, NM, SP, UN-N, and KS reviewed the data and the manuscript. All authors gave final approval for publication.

## Conflict of Interest

The authors declare that the research was conducted in the absence of any commercial or financial relationships that could be construed as a potential conflict of interest.

## Footnotes

1 http://www.illumina.com/products/cbot.html

2 http://blast.ncbi.nlm.nih.gov/Blast.cgi

## Supplementary Material

Supplementary Table 1 Chromosomal locations for restriction fragment presence/absence (PA) loci of African catfish (*Clarias gariepinus*) BLAST in Japanese rice fish (*Oryzias latipes*), zebrafish (*Danio rerio*), Japanese pufferfish (*Takifugu rubripes*) and chicken (*Gallus gallus*) genomes (30:70, male:female) (ZZ/ZW sex-determination type).

Supplementary Table 2 Gene function and pathway for restriction fragment presence/absence (PA) loci of African catfish (*Clarias gariepinus*) from a BLAST search of Japanese rice fish (*Oryzias latipes*), Japanese pufferfish (*Takifugu rubripes*), zebrafish (*Danio rerio*) and chicken (*Gallus gallus*) genome (30:70, male:female) (ZZ/ZW sex-determination type).

Supplementary Table 3 Repeat search for restriction fragment presence/absence (PA) loci of African catfish (*Clarias gariepinus*) (30:70, male:female) (ZZ/ZW sex-determination type).

Supplementary Table 4 Gene function and pathway for restriction fragment presence/absence (PA) loci of African catfish (*Clarias gariepinus*) from a BLAST search of Japanese rice fish (*Oryzias latipes*), Japanese pufferfish (*Takifugu rubripes*), zebrafish (*Danio rerio*) and chicken (*Gallus gallus*) genome (70:30, male:female) (XX/XY sex-determination type).

Supplementary Table 5 Repeat search for restriction fragment presence/absence (PA) loci of African catfish (*Clarias gariepinus*) (70:30, male:female) (XX/XY sex-determination type).

Supplementary Table 6 Chromosomal locations for restriction fragment presence/absence (PA) loci of African catfish (*Clarias gariepinus*) BLAST in Japanese rice fish (*Oryzias latipes*), zebrafish (*Danio rerio*), Japanese pufferfish (*Takifugu rubripes*) and chicken (*Gallus gallus*) genomes (70:30, male:female) (XX/XY sex-determination type).

Supplementary Table 7 Homology for restriction fragment presence/absence (PA) loci of African catfish (*Clarias gariepinus*) from a BLAST search of Japanese rice fish (*Oryzias latipes*), Japanese pufferfish (*Takifugu rubripes*), zebrafish (*Danio rerio*) and chicken (*Gallus gallus*) genome (30:70, male:female) (ZZ/ZW sex-determination type).

Supplementary Table 8 Homology for restriction fragment presence/absence (PA) loci of African catfish (*Clarias gariepinus*) from a BLAST search of Japanese rice fish (*Oryzias latipes*), Japanese pufferfish (*Takifugu rubripes*), zebrafish (*Danio rerio*) and chicken (*Gallus gallus*) genome (70:30, male:female) (XX/XY sex-determination type).
