# Supplementary material for: An Investigation of ZZ/ZW and XX/XY Sex Determination Systems in North African Catfish (Clarias gariepinus, Burchell, 1822)
Source: Front Genet. 2021 Jan 5;11:562856. doi: 10.3389/fgene.2020.562856 (PMC7874028; doi:10.3389/fgene.2020.562856)
Supplement: Supplementary Table 1 — Chromosomal locations for restriction fragment presence/absence (PA) loci of African catfish (Clarias gariepinus) BLAST in Japanese rice fish (Oryzias latipes), zebrafish (Danio rerio), Japanese pufferfish (Takifugu rubripes) and chicken (Gallus gallus) genomes (30:70, male:female) (ZZ/ZW sex-determination type). [file Data_Sheet_1.docx]

**Supplementary Table S1**│Chromosomal locations for restriction fragment presence/absence (PA) loci of African catfish (*Clarias gariepinus*) BLAST in Japanese rice fish (*Oryzias latipes*), zebrafish (*Danio rerio*), Japanese pufferfish (*Takifugu rubripes*) and chicken (*Gallus gallus*) genomes (30:70, male:female) (ZZ/ZW sex-determination type).

| Locus id | *Oryzias latipes* | *Danio rerio* | *Takifugu rubripes* | *Gallus gallus* |
| --- | --- | --- | --- | --- |
| PA51665230 | 10 | 14 | 14 | - |
| PA51673180 | 15 | 8 | 15 | - |
| PA51669555 | 12 | 1 | - | - |
| PA51635654 | 16 | - | - | - |
| PA51636349 | 12 | - | - | - |
| PA51655919 | 10 | - | 14 | - |
| PA51667023 | 19 | 1 | - | - |
| PA51671027 | 15 | 16 | 21 | - |
| PA51662083 | - | - | 9 | - |

**Supplementary Table S2│**Gene function and pathway for restriction fragment presence/absence (PA) loci of African catfish (*Clarias gariepinus*) from a BLAST search of Japanese rice fish (*Oryzias latipes*), Japanese pufferfish (*Takifugu rubripes*), zebrafish (*Danio rerio*) and chicken (*Gallus gallus*) genome (30:70, male:female) (ZZ/ZW sex-determination type).

| Locus id | Gene^1,2^ | Product | Function | Component | Pathway | Reference |
| --- | --- | --- | --- | --- | --- | --- |
| PA51665230 | ***PCDH2AB3*** | Protocadherin alpha subfamily C, 2 | Calcium ion binding | Plasma membrane | Cell adhesion, homophilic cell adhesion via plasma membrane adhesion molecules | Gaudet et al. (2011) |
| PA51673180 | ***DCTN4*** | Dynactin subunit 4 | Protein N-terminus binding | Centrosome, nucleus, cytosol | Antigen processing and presentation of exogenous peptide antigen via MHC class II, endoplasmic reticulum to Golgi vesicle-mediated transport | Lim et al. (2006) |
|  |  |  |  |  |  |  |

*^1^All genes show sequence similarity with partial sequence of DArT loci more than 50% from database. ^2^All genes show an E-value with partial sequence of DArT loci lower than 0.05 from database.*

Gaudet, P., Livstone, M. S., Lewis, S. E., and Thomas, P. D. (2000). Phylogenetic-based propagation of functional annotations within the gene ontology consortium. *J. Biol. Chem.* 275, 4834–4839. doi: [10.1074/jbc.275.7.4834](https://doi.org/10.1074/jbc.275.7.4834)

Lim, C. M., Cater, M. A., Mercer, J. F., and La Fontaine, S. (2006). Copper-dependent interaction of dynactin subunit p62 with the N terminus of ATP7B but not ATP7A. *J. Biol. Chem.* 281, 14006–14014. doi: 10.1074/jbc.M512745200

**Supplementary Table S3│**Repeat searched for restriction fragment presence/absence (PA) loci of African catfish (*Clarias gariepinus*) (30:70, male:female) (ZZ/ZW sex-determination type).

| Repeat | Type | PA loci |
| --- | --- | --- |
| LTR Retrotransposon | Gypsy | 3 |
| Non-LTR Retrotransposon | Rex1 | 1 |

**Supplementary Table S4│**Gene function and pathway for restriction fragment presence/absence (PA) loci of African catfish (*Clarias gariepinus*) from a BLAST search of Japanese rice fish (*Oryzias latipes*), Japanese pufferfish (*Takifugu rubripes*), zebrafish (*Danio rerio*) and chicken (*Gallus gallus*) genome (70:30, male:female) (XX/XY sex-determination type).

| Locus id | Gene^1,2^ | Product | Function | Component | Pathway | Reference |
| --- | --- | --- | --- | --- | --- | --- |
| PA51633768 | ***GUCD1*** | Guanylyl cyclase domain-containing 1 | Ubiquitous expression in lung, liver | Nucleoplasm | Liver regeneration | Bellet et al. (2014) |
| PA51665576 | ***ADD3*** | Adducin 3 | Plays a role in actin filament capping | Cytoskeleton, cytosol, condensed nuclear chromosome | Actin filament bundle assembly, barbed-end actin filament capping, transmembrane transport | Kruer et al. (2013) |
| PA51658745 | ***DTNA*** | Dystrobrevin alpha | PDZ domain binding, zinc ion binding | Plasma membrane | Chemical synaptic transmission, neuromuscular synaptic transmission, Signal transduction, Striated muscle contraction | Sadoulet-Puccio et al. (1996) |

*^1^All genes show sequence similarity with partial sequence of DArT loci more than 50% from database. ^2^All genes show an E-value with partial sequence of DArT loci lower than 0.05 from database.*

Bellet, M. M., Piobbico, D., Bartol, D., Castelli, M., Pieroni, S., Brunacci, C., et al. (2014). *NEDD4* controls the expression of *GUCD1*, a protein upregulated in proliferating liver cells. *Cell Cycle* 13, 1902–1911. doi: 10.4161/cc.28760

Kruer, M. C., Jepperson, T., Dutta, S., Steiner, R. D., Cottenie, E., Sanford, L., et al. (2013). Mutations in γ adducin are associated with inherited cerebral palsy. *Ann. Neurol.* 74, 805–814. doi: 10.1002/ana.23971

Sadoulet-Puccio, H. M., Khurana, T. S., Cohen, J. B., and Kunkel, L. M. (1996). Cloning and characterization of the human homologue of a dystrophin related phosphoprotein found at the Torpedo electric organ post-synaptic membrane. *Hum. Mol. Genet.* 5, 489–496. doi: [10.1093/hmg/5.4.489](https://doi.org/10.1093/hmg/5.4.489)

**Supplementary Table S5**│Repeat searched for restriction fragment presence/absence (PA) loci of African catfish (*Clarias gariepinus*) (70:30, male:female) (XX/XY sex-determination type).

| Repeat | Type | PA loci |
| --- | --- | --- |
| DNA transposon | Mariner/Tc1 | 3 |
| LTR Retrotransposon | Gypsy | 3 |
| Non-LTR Retrotransposon | SINE | 1 |

**Supplementary Table S6│**Chromosomal locations for restriction fragment presence/absence (PA) loci of African catfish (*Clarias gariepinus*) BLAST in Japanese rice fish (*Oryzias latipes*), zebrafish (*Danio rerio*), Japanese pufferfish (*Takifugu rubripes*) and chicken (*Gallus gallus*) genomes (70:30, male:female) (XX/XY sex-determination type).

| Locus id | *Oryzias latipes* | *Danio rerio* | *Takifugu rubripes* | *Gallus gallus* |
| --- | --- | --- | --- | --- |
| PA51670815 | 3 | 1 | 11 | - |
| PA51665576 | 1 | 22 | - | - |
| PA51669699 | - | - | 16 | - |
| PA51637272 | 22 | 11 | 1 | - |
| PA51619964 | 24 | 5 | 20 | - |
| PA51669858 | 10 | - | - | - |
| PA51657618 | - | - | 22 | - |
| PA51642594 | - | - | - | Z |
| PA51620163 | 4 | 3 | 19 | - |
| PA51633768 | 23 | 4 | 7 | - |
| PA51671566 | 7 | - | 16 | - |
| PA51669403 | 1 | 24 | - | - |
| PA51657744 | 6 | 13 | - | - |

**Supplementary Table S7│**Homology for restriction fragment presence/absence (PA) loci of African catfish (*Clarias gariepinus*) from a BLAST search of Japanese rice fish (*Oryzias latipes*), Japanese puffer (*Takifugu rubripes*), zebrafish (*Danio rerio*) and chicken (*Gallus gallus*) genome (30:70, male:female) (ZZ/ZW sex-determination type).

| Locus id | Gene^1,2^ | Species | Region | Reference |
| --- | --- | --- | --- | --- |
| PA51673180 | ***DCTN4*** | *Ornithorhynchus anatinus* | X1 chromosome, CDS | Warren et al. (2008) |

*^1^All genes show sequence similarity with partial sequence of DArT loci more than 50% from database. ^2^All genes show an E-value with partial sequence of DArT loci lower than 0.05 from database.*

**Supplementary Table S8│**Homology for restriction fragment presence/absence (PA) loci of African catfish (*Clarias gariepinus*) from a BLAST search of Japanese rice fish (*Oryzias latipes*), Japanese puffer (*Takifugu rubripes*), zebrafish (*Danio rerio*) and chicken (*Gallus gallus*) genome (70:30, male:female) (XX/XY sex-determination type).

| Locus id | Gene^1,2^ | Species | Region | Reference |
| --- | --- | --- | --- | --- |
| PA51633768 | ***GUCD1*** | *Danaus plexippus plexippus* | Z chromosome, CDS | Gu et al. (2019) |
|  |  | *Cynoglossus semilaevis* | Z chromosome, CDS | Chen et al. (2014) |
|  |  | *Drosophila busckii* | X chromosome, CDS | Renschler et al. (2019) |
|  |  | *Canis lupus familiaris* | X chromosome, CDS | Lindblad-Toh et al. (2005) |

*^1^All genes show sequence similarity with partial sequence of DArT loci more than 50% from database. ^2^All genes show an E-value with partial sequence of DArT loci lower than 0.05 from database*

Chen, S., Zhang, G., Shao, C., Huang, Q., Liu, G., Zhang, P., et al. (2014). Whole-genome sequence of a flatfish provides insights into ZW sex chromosome evolution and adaptation to a benthic lifestyle. *Nat. Genet*. 46, 253–260. doi: 10.1038/ng.2890

Gu, L., Reilly, P. F., Lewis, J. J., Reed, R. D., Andolfatto, P., and Walters, J. R. (2019). Dichotomy of dosage compensation along the neo Z chromosome of the Monarch butterfly. *Curr. Biol*. 29, 4071–4077. doi: 10.1016/j.cub.2019.09.056.

Renschler, G., Richard, G., Valsecchi, C. I. K., Toscano, S., Arrigoni, L., Ramirez, F., et al. (2019). Hi-C guided assemblies reveal conserved regulatory topologies on X and autosomes despite extensive genome shuffling. *Genes Dev*. 33, 1591–1612. doi: 10.1101/gad.328971.119.

Lindblad-Toh, K., Wade, C. M., Mikkelsen, T. S., Karlsson, E. K., Jaffe, D. B., Kamal, M., et al. (2005). Genome sequence, comparative analysis and haplotype structure of the domestic dog. *Nature* 438, 803–819. doi: 10.1038/nature04338
